# The Fossil Lithistida Collection at the Natural History Museum, London (UK)

**DOI:** 10.3897/BDJ.10.e87106

**Published:** 2022-08-24

**Authors:** Consuelo Sendino, Andrew Tucker

**Affiliations:** 1 Natural History Museum, London, United Kingdom Natural History Museum London United Kingdom

**Keywords:** Fossil Lithistida Collection, stratigraphic range, geographic range, taxonomic identification, digitisation, historical collections, Natural History Museum

## Abstract

**Background:**

This paper presents a quantitative and detailed description of the Fossil Lithistida Collection in the Natural History Museum, London. This collection started to be built with the first fossil sponges from the Cretaceous of Wiltshire, collected by William Smith in 1816 and 1818 for the first geological map of England. The latest specimen to enter the collection was collected from the Permo-Carboniferous of Norway by Angela Milner, a researcher at the Museum, in 2000. Although they are mostly from the Cretaceous of England, lithistids are represented from the Cambrian to Cenozoic of England. This makes this collection key for studying this group. Lithistid study will help with understanding of biosilicification evolution in sponges to unlock the changing patterns in the silica cycle in the oceans through geological time.

**New information:**

A dataset with information about all the Fossil Lithistida Collection is available through the NHM Data Portal and Suppl. material 1. This dataset includes taxonomic identifications, registration numbers of the specimens, geographic and stratigraphic details, information about specimen collectors and donors, type status and publications where the specimens have been referred.

## Introduction

**Figure 1.**
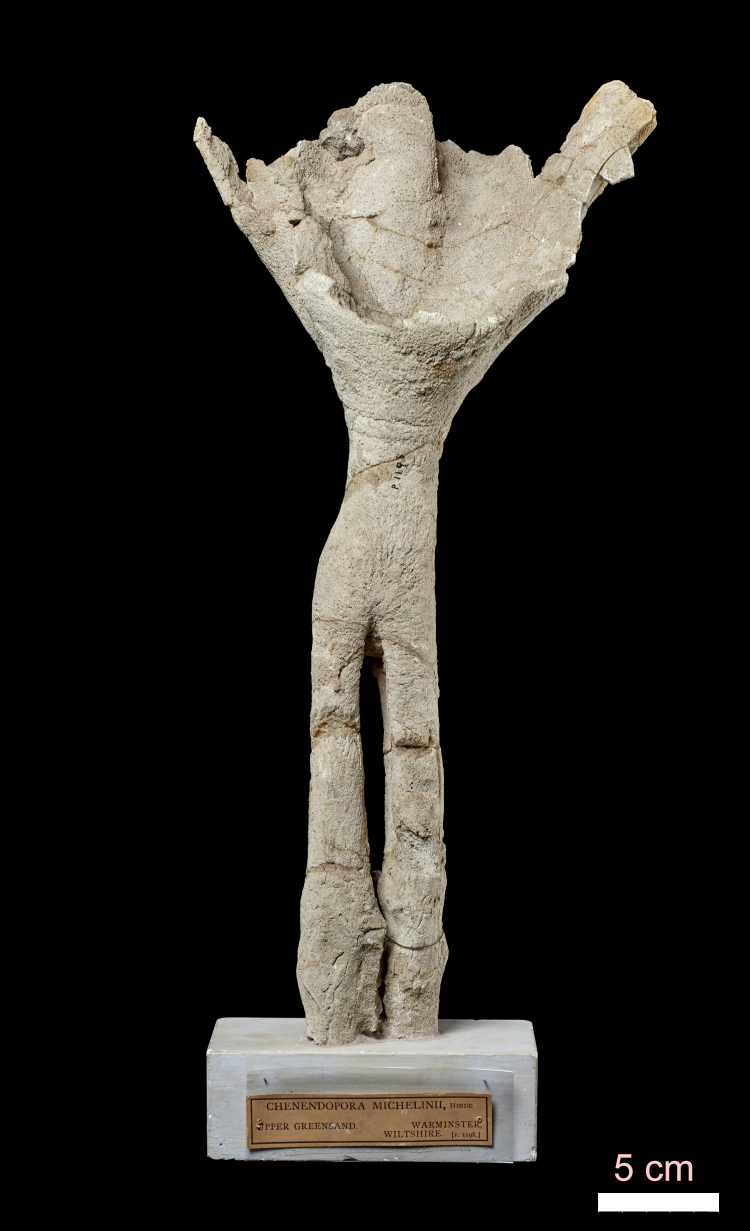
Specimen NHM UK PI P 1198 (1) of *Chenendoporamichelinii* Hinde, 1884. PARATYPE. Cretaceous of Warminster, Wiltshire, England. Lower portion of the stem is divided into root-like extensions.

Amongst siliceous sponges, demosponges are the most successful, possessing different types of skeletons. The most heavily silicified sponges are known as ‘lithistids’, a polyphyletic group which have inhabited the Earth for more than 513 Ma. They are commonly called stony sponges in recognition of their solid skeletons, in contrast to other spicule-bearing sponges and even spicule-lacking sponges that are largely compressible. Lithistids have solid silica skeletons with mainly articulating choanosomal megascleres, desmas that form a coherent skeletal framework. During the Palaeozoic and Mesozoic, they inhabited shallower waters with higher silica contents than today (Pisera 2004). Therefore, the most common lithistids in the collection, from the English Cretaceous, used to live on soft muddy substrates (the Chalk) not in hard substrates or firm, rocky sea-beds as they do today. This could be the cause for some lithistids developing a stalk to attach to the muddy substrate (Zittel 1878; Fig. 1). On the other hand, they were reef-like constructors, mainly during the Jurassic (Pisera 2004). Probably the silica content in seawater was higher during Palaeozoic and Mesozoic (Muir et al. 2017) than in the Cenozoic.

The Fossil Lithistida Collection at the Natural History Museum (NHM) contains 5088 hand specimens and 264 thin sections mainly from the Cretaceous of the United Kingdom and Germany. These specimens have been digitised on Excel, on a template that is compatible with Emu (Sendino 2009), the collection management system used at the NHM and the raw data have been uploaded on the NHM Data Portal (https://data.nhm.ac.uk/dataset/the-nhm-fossil-lithistida-collection) and here (Suppl. material 1). Of these specimens, almost 600 have been already published in 38 publications, mainly by George J. Hinde, Anton Schrammen, Filip Počta and William Sollas (see Suppl. material 2), of which 398 are type and figured specimens. This is a comprehensive dataset with reference to updated taxonomic names, geographic and stratigraphic data, donors and bibliographical references where they have been published.

**Figure 2.**
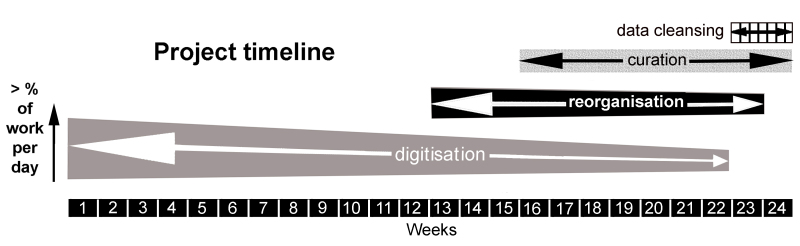
Distribution of the project timeline.

The digitisation of these lithistid specimens was carried out in an internal NHM project over six months (Fig. 2) which also included the curation of the specimens, re-boxing them with acid-free trays and placing plastazote to protect most of the specimens, as there was not enough time to do it for all of them. The reason for this is that the number of the specimens was higher than expected. Resources included recruitment of an assistant curator and four volunteers, use of acid free trays and plastazote foam.

## General description

### Purpose

The purpose of the digitisation of this collection, as part of the NHM Science Strategy, is securing the future of the collection making it accessible and digitally available, also engaging and involving the widest possible audience, reaching out nationally and globally, onsite and online. Most of the specimens were curated to the highest curation standards, replacing trays with acid-free ones for all the specimens and re-boxing them with plastazote for half of the specimens.

### Additional information

The curation and digitisation of this collection was funded by Museum internal funding. For this, a project was created estimating 3,000 specimens in this collection, being aware of the average number of specimens per drawer with fossil lithistids. The complete digitisation of the specimens resulted in 40% more specimens. Sometimes, the drawers had specimens which were not lithistids and were relocated to the right place. There were locations with a mixture of specimens (specimens for exhibitions, used in tours and/or student classes) where we had to discern which ones were lithistids and which ones belonged to other groups to relocate them.

There were 337 specimens without any registration number. We generated these and created the labels for these specimens and 400 further specimens which had only a yellow sticker with the registration number. We included all the labels in special transparent archival polyester sleeves and printed the new labels generated on special archival paper with archival ink.

The digitisation took up 62.5% of the time and, as the project timeline advanced, digitisation, reorganisation and curation were combined. To finish with data cleansing, the last two weeks were shared with curation and reorganisation of some specimens.

The digitisation involved recording all the written information documented on the specimen labels and/or the catalogue books when the specimens did not have associated labels or the data were incomplete. We also studied those publications where the specimens were cited, described and/or figured. For this, an Excel template compatible with Emu was built. In order to make sure about digitising lithistid taxa, we followed the Porifera Treatise (Finks et al. 2004) considering the Subclass Lithistida Schmidt, 1870 and papers on fossil lithistids. At the same time as the specimens were digitised, the taxonomic names were updated, by batches, after the most updated revisions. In the case of geographic and stratigraphic records, they were updated when they were recorded. Quality control and assurance procedures were implemented at all stages to avoid errors and repeating them.

Once all the data were recorded, the last few weeks were used for cleansing and standardising the data, including standardisation of acquisition details and their completeness, with the help of NHM books on donations/acquisitions and the *World Palaeontological Collections* book by Cleevely (1983). This stage helped us to correct the mistakes made.

The reorganisation of those sponges which were non-lithistids in their corresponding locations was carried out when we had most of the specimens databased and lasted half of the project timeline. The more specimens digitised, the easier it was to recognise those non-lithistid taxa and also find those missing specimens in the lithistid NHM locations. The non-lithistids were relocated to the right places in the Fossil Porifera Collection. Concerning the missing specimens, some appeared in other adjacent drawers or in drawers with mixed specimens from the collections used for students or for exhibitions. 200 missing lithistids were recorded on the dataset as the trays contained the labels, but not the specimens. Most of these specimens were found (90%) in other locations of the Porifera Collections.

Curation, including re-boxing with plastazote and acid free trays, was done with the help of four volunteers who worked at the same time as the collections were reorganised, digitised and the data cleansing done.

The project timeline prepared before the project execution was very useful to focus the project and take decisions.

A further stage in the digitisation of this collection will be to take images of those specimens which have not been imaged previously. This will help stakeholders with research and identification of lithistid specimens.

**History of the Collection**

**Figure 3.**
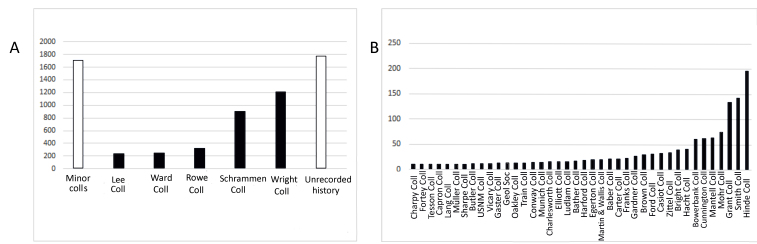
Origin of the NHM Fossil Lithistida Collection. A. The largest collections; B. Those minor collections shown in A.

This collection is compiled by purchases, donations and bequests of historical collectors, researchers and, more recently, NHM staff. About 37% of the collection has unrecorded history, but the remainder is mainly made up of small collections of less than 200 specimens (Fig. 3B) and from the largest to the smallest collections: the Claud William Wright Collection from the Cretaceous of England, donated between 1948 and 1949; the Anton Schrammen Collection from the Palaeozoic and Mesozoic of Sweden, Canada, USA, Germany, Poland, purchased between 1904 and 1938; the Arthur Walter Rowe Collection from the Cretaceous of England, purchased in 1926; the David J. Ward Collection from the Cretaceous of England donated in 1994; and the John Edward Lee Collection, from the Cretaceous and Silurian of different countries (England and Wales, UK; Germany; Canada and Sweden) presented in 1885 (Fig. 3A).

The importance of this collection lies not only in its stratigraphic and geographic range, but also in the scientific value of its types and figured specimens to species and even subspecies/varieties that have not been revised since their original descriptions (126 taxa) (Suppl. material 2). Currently, there are taxa not recognised for the specimens identified to subspecies and variety levels. Recent studies have focused on biosilicification evolution in lithistids which may help to unlock the changing patterns in the silica cycle in the oceans through geological time (Conley et al. 2017).

## Project description

### Title

Digitisation of the Lithistida Collection

### Personnel

An assistant curator and four volunteers to help with curation.

### Study area description

Those specimens kept at the NHM belonging to the sponge Subclass Lithistida Schmidt, 1870, for which we followed the Porifera Treatise (Finks et al. 2004) and papers on fossil lithistids.

### Design description

The project was created estimating a number of specimens, being aware of the average number of specimens per drawer with fossil lithistids. For this, a project timeline was created (Fig. 2).

### Funding

Natural History Museum internal funding for 2021 (DIF bid number 490).

## Geographic coverage

### Description

**Figure 4.**
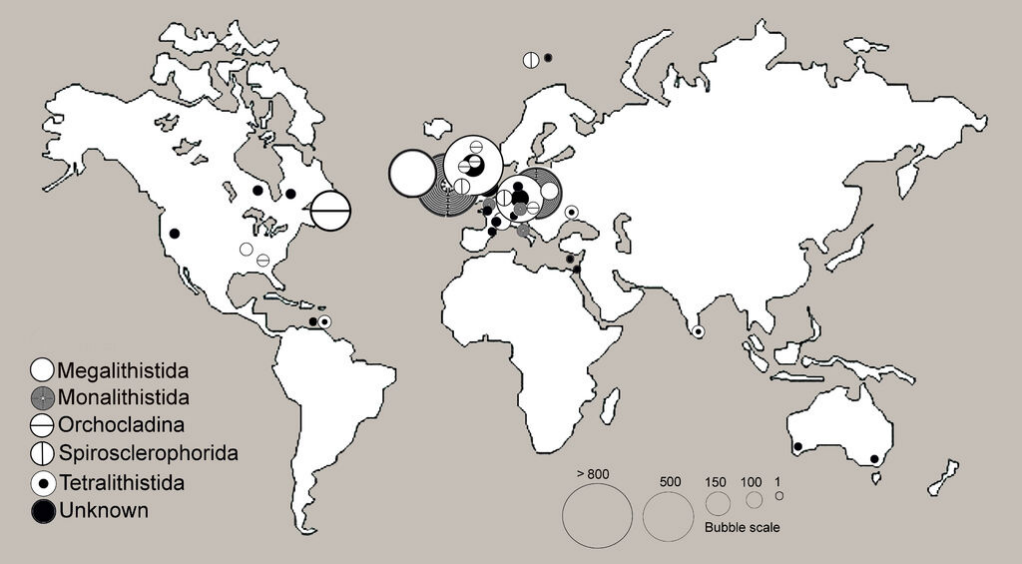
Bubble map with fossil sponge sites from where the NHM keeps specimens.

Most of the Collection comes from the UK (61%), mainly from England (Fig. 4). We have included those which were of doubtful origin with the others as the percentage of those in doubt is less than 1% in most cases. Those taxa where it was impossible to find out the updated taxonomic names have been included under ‘Unknown’ (order) on the map as those specimens and their taxonomic names need revision. We have to highlight that most of the thin sections are of megalithistids from the Cretaceous of Germany and monalithistids from the Jurassic of Poland. These sections form a good resource for their study.

As we can see on the map, most of the specimens come from Europe and North America and belong to the Treatise orders of megalithistids, monalithistids, spirosclerophorids, tetralithistids and orchocladids. The fact that most studied continents are Europe and North America is due to the collections being mainly historical and having been collected in the 19^th^ and 20^th^ centuries, when fieldwork was done in the researchers’ countries and on expeditions. This creates a bias in the results that is well observed, in general, in palaeontology of invertebrates in all worldwide museums.

## Taxonomic coverage

### Description

**Figure 5.**
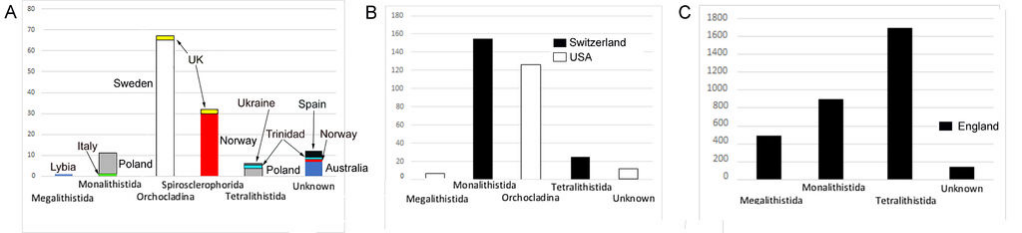
Number of specimens by order and country. A, B and C have different scales due to the difference in their abundance. A. Orders with less than 70 specimens per country; B. Countries with specimens between 100 and 200 specimens; C. England, UK with most of the specimens.

This collection includes 406 taxa, of which there are 338 species and 15 varieties that are distributed mainly amongst tetralithistids, monalithistids, megalithistids, orchocladinids and spirosclerophorids (Figs 4, 5 and Suppl. material 1). To know more about these orders, see Finks et al. (2004).

Tetralithistids are the most common, 44% of the collection, with representation in Central Europe, Ukraine, India, Republic of Trinidad and Tobago and mainly in UK-England. The next most common are the monalithistids (32%) with representation in Central Europe and UK-England as well. In lesser proportion, megalithistids (12%) are represented in Central Europe, Libya, UK-England and USA. Orchocladids (8%) have been collected in Europe, UK and USA. Finally, the spirosclerophorids (1%) have been found in Europe and UK-Wales and axinellids with a few specimens. Four percent of the collection have not been possible to include in an order.

## Temporal coverage

### Notes

**Figure 6.**
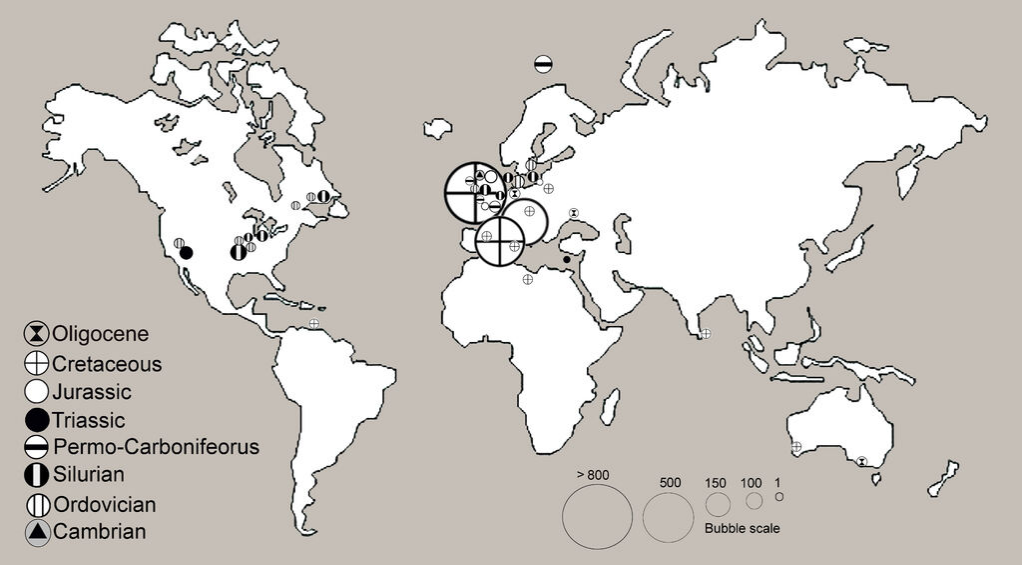
Bubble map with fossil sponge sites by stratigraphy from specimens kept at the NHM.

The stratigraphic distribution plays an important role in this fossil collection. This is linked to the origin. As most of the collection comes from the UK, most is Cretaceous (81%). Other localities where the Cretaceous lithistids have representation in this Collection are in Europe, Australia, India, Libya and the Republic of Trinidad and Tobago. Ten percent is Jurassic, coming from Europe and mainly from UK-England. Silurian lithistids (5%) are mainly from Sweden and North America. In much less proportion is the Ordovician (2%) of Europe and North America. The Cambrian, Permo-Carboniferous, Triassic and Paleogene lithistids are represented scarcely from sites in Europe, Ukraine, Australia, Cyprus, Israel, UK (England, Wales and Scotland) and USA (Fig. 6).

## Collection data

### Collection name

The NHM Fossil Lithistida Collection

### Collection identifier

Fossil Lithistida Collection

### Parent collection identifier

Demospongiae

### Specimen preservation method

Isolated, mounted dried specimens and thin sections

### Curatorial unit

Palaeobiological collections, sponges

## Usage licence

### Usage licence

Creative Commons Public Domain Waiver (CC-Zero)

## Data resources

### Data package title

The NHM Fossil Lithistida Collection

### Resource link

https://data.nhm.ac.uk/dataset/the-nhm-fossil-lithistida-collection

### Number of data sets

1

### Data set 1.

#### Data set name

Fossil Lithistida Collection

#### Data format

CSV

#### Download URL

https://data.nhm.ac.uk/dataset/the-nhm-fossil-lithistida-collection/resource/81382c9e-6d16-4cdf-ae97-139d7d6787b8

#### Description

CSV database with specimen information of NHM Fossils Lithistida Collection

**Data set 1.** 

Column label	Column description
ID	ID number of the dataset
Registration Number	Registration Number
Number of items with the same registration number	Number of items with the same registration number
Type of specimen	If it is hand specimen or thin section
Individual Description	Description
Previous registration number	Previous registration numbers from the collector or other museum
Type of previous registration	Collector or museum
Taxon	Taxonomic name, including open nomenclature
Figured/ Type/Referred	If the specimen has been figured, referred or it is a type
Site	Geographic details
Stratigraphy	Stratigraphic details
Acquisition Source	Acquisition party/person details
Acquisition Method	If the specimen was donated, purchased or bequeathed
Publication1	Publication where the specimen has been referred
Publication2	Publication where the specimen has been referred
Publication3	Publication where the specimen has been referred

## Additional information

The Lithistida Collection has been reorganised, digitised with mainly updated taxonomic names and curated to high standards according to the Museum curation protocols, with acid-free trays for all the specimens and plastazote for half of the specimens. This collection is ready for use for stakeholders and also prepared for its move to the Thames Valley Science Park, the new NHM science and digitisation centre where the Porifera Collection will be moved.

## Supplementary Material

BED76373-8328-54E8-98B9-B076C65074CA 10.3897/BDJ.10.e87106.suppl1 Supplementary material 1 Fossil Lithistida Collection Dataset Data type Taxonomy, sites, stratigraphy Brief description Taxonomy, sites, stratigraphy and acquisition details of the fossil lithistid specimens at the NHM. File: oo_715469.csv https://binary.pensoft.net/file/715469 Andrew Tucker; Consuelo Sendino

FE0DA6AB-2098-5E1B-8CE8-039BD7CCC729 10.3897/BDJ.10.e87106.suppl2 Supplementary material 2 Bibiliographic references for NHM fossil lithistid type, figure and cited specimens Data type References for type, figure and cited specimens Brief description A comprehensive list of bibliographic references where the NHM Fossil Lithistida Collection has been published. File: oo_689081.pdf https://binary.pensoft.net/file/689081 Consuelo Sendino
