# Supplementary material for: The Fossil Lithistida Collection at the Natural History Museum, London (UK)
Source: Biodivers Data J. 2022 Aug 24;10:e87106. doi: 10.3897/BDJ.10.e87106 (PMC9848467; doi:10.3897/BDJ.10.e87106)
Supplement: Supplementary material 2 — Bibiliographic references for NHM fossil lithistid type, figure and cited specimens [file bdj-10-e87106-s002.pdf]

APPENDIX:

REFERENCES FOR LITHISTIDA TYPE, FIGURE & CITED SPECIMENS

Billings, E. 1865. On some new or little-known species of lower Silurian fossils from Potsdam Group (Primordial Zone). In *Palaeozoic Fossils, vol. 1. Containing Descriptions and Figures of New or Little Known Species of Organic Remains from the Silurian Rocks*. Geological Survey of Canada. Baillière. London, New York, & Paris, 426 pp.

Blue Circle Cement Organization. 1948. Flints. The Blue Circle. *House Journal of the Blue Circle Cement Organization*, 2: 10-13.

Castell, C.P., Cox, L.R. & Muir-Wood, H.M. 1962. *British Mesozoic Fossils*. British Museum Natural History. London, 205 pp.

Dixon, F. 1850. *The geology and fossils of the Tertiary and Cretaceous formations of Sussex*. London, 423 + xvi pp.

Donovan, S.K. 2000. Fossils explained 31: Sponges 1. *Geology Today*, 16: 194-198.

Hinde, G.J. 1880. *Fossil Sponge Spicules: From the Upper Chalk. Found in the Interior of a Single Flint-Stone From Horstead in Norfolk*. Phil. Diss Munich, Wolf, 81 pp.

Hinde, G.J. 1882. Notes on Fossil Calcispongiae, with Descriptions of New Species. *Annals and Magazine of Natural History*, Series 5, 10: 185-206.

Hinde, G.J. 1884. *Catalogue of the Fossil Sponges in the Geological Department of the British Museum (Natural History). With descriptions of new and little-known species*. BM(NH), London, 248 pp.

Hinde, G.J. 1886. On beds of sponge-remains in the Lower and Upper Greensand of the South of England. *Philosophical Transactions of the Royal Society of London*, 176 (2) : 403-453, pls 40-45.

Hinde, G.J. 1888. On the Chert and Siliceous Schists of the Permo-Carboniferous Strata of Spitzbergen, and on t he Characters of the Sponges therefrom, whi ch have been described by Dr. E. Dunikowski . Geolological Magazine, London, (3) 5: 241- 251.

Hinde, G.J. 1893. *The British fossil sponges. Part III. Sponges of Jurassic strata*. Palaeontographical Society Monograph, London, 189—254.

Lee, J.E. 1839. Notice of undescribed zoophytes from the Yorkshire Chalk. *Magazine of Natural History*, New Series, 3: 10-17.

Lewis, C. 2017. *The Enlightened Mr. Parkinson: The Pioneering Life of a Forgotten English Surgeon*. Icon Books, London, 320 pp.

Lewis, D.N. 2000. Fossils Explained 30: Macrofossils in flint. *Geology Today*, 16: 153-158.

Mantell, G.A. 1822. The Fossils of the South Downs; or illustrations of the geology of Sussex. London, 327 pp.

Oakley, K.P. 1937. Cretaceous sponges: some biological and geological considerations. *Proceedings of the Geologists' Association*, 48: 330-348.

Oakley, K.P. 1942. Flexible sponges of Oligocene age from Ukraine. *Proceedings of the Geologists' Association*, 53: 106-107.

Parkinson, J. 1808. *Organic Remains of a Former World. An examination of the mineralized remains of the Vegetables and Animals of the antediluvian world, generally termed extraneous fossils*. Volume 2, London, 286 pp.

Pisera, A. 2000. New species of lithistid sponges from the Paleogene of the Ukraine. *Zoosystema*, 22: 285-298.

Počta, P. 1907. Sur quelques éponges du Sénonien de Nice. *Bulletin de la Société Géologique de la France*, Series 4, 7: 163-173.

Richardson, L. & Thacker, A.G. 1920. On the stratigraphical and geographical distribution of the sponges of the Inferior Oolite of the West of England. *Proceedings of the Geologists' Association*, 31: 161-186.

Rigby, J.K. & Scrutton, C.T. 1985. Sponges, chaetetids and stromatoporoids. In Murray, J.W. (ed.), *Atlas of Invertebrate Macrofossils*. Longman, London, pp. 3-10.

Salter, J.W. 1860. Note on the fossils from Spitzbergen. *In* Lamont, J., Notes about Spitzbergen in 1859 (with Appendix). *Quarterly Journal of the Geological Society of London*, 16: 439-442.

Schrammen, A. 1901. Neue Kieselschwämme aus der oberen Kreide der Umgebung von Hannover und von Hildesheim. *Mitteilungen aus dem Roemer-Museum*, 14: 1-26.

Schrammen, A. 1910. Die Kieselspongien der oberen Kreide von Nordwestdeutschland. I. Teil. Tetraxonia, Monaxonia und Silicea incert. sedis. *Palaeontographica*, Supplement 5: 1-175.

Schrammen, A. 1924. Die Kieselspongien der oberen Kreide von Nordwestdeutschland. III. und letzter Teil. *Monographien zur Geologie und Palaeontologie*, Serie 1, 2: 159 pp.

Schrammen, A. 1924. Zur Revision der Jura-Spongian von Süddeutschland. Jahresbericht und Mitteilungen des Oberrheinischen Geologischen Vereins, New series, 13: 125-154.

Sendino, C. 2020. The Natural History Museum Fossil Porifera Collection. *Collections: A Journal for Museum and Archives Professionals*, 16(4): 363–380. DOI: 10.1177/1550190620964042

Smith, W. 1816-1819. *Strata identified by Organized Fossils, containing prints on cloured paper of the most characteristic specimens in each Stratum*. London, 32 pp.

Smith, W. 1817. *Stratigraphical system of organized Fossils. With reference to the specimens of the original geological collection in the British Museum, explaining their state of preservation and their use in identifying the British Strata*. London, xi + 118 + [3] pp.

Sollas, W.J. 1877. On the structure and affinities of the genus *Siphonia*. *Quarterly Journal of the Geological Society of London*, 33: 790-834.

Sollas, W.J. 1883. Descriptions of Fossil Sponges from the Inferior Oolite, with a notice of some from the Great Oolite. *Quarterly Journal of the Geological Society of London*, 39: 541-554.

Sollas, W.J. 1885. On an hexactinellid sponge from the Gault and a lithistid from the Lias of England. *Scientific Proceedings of the Royal Dublin Society,* New series, 4: 443-446.

Taylor, P.D. 1990. Eyewitness Guides. Fossil. London, 64 pp.

Thomas, H.D. 1935. On some sponges and a coral of Upper Cretaceous age from Toco Bay, Trinidad. *Geological Magazine*, 72: 175-179.

Walker, C. & Ward, D. 1992. Fossils (Eyewitness Handbook). Dorling Kindersley, London, 320 pp.

Woodward, S.P. 1864. On the Nature and Origin of Banded Flints. *Geological Magazine*, Series 1, 1: 145-149.

Woodward, H.B. 1906. Excursion to Lyme Regis, Easter, 1906. *Proceedings of the Geologists' Association,* 19: 320-340.
